# Ligand-Induced Synthesis of Highly Stable NM88(DB)@COF-JLU19 Composite: Accelerating Electron Flow for Visible-Light-Efficient Degradation of Tetracycline Hydrochloride

**DOI:** 10.3390/polym16040539

**Published:** 2024-02-17

**Authors:** Jinxia Zhao, Jingchao Liu, Zenghe Li, Yilin Yin

**Affiliations:** 1 College of Chemistry, Beijing University of Chemical Technology, Beijing 100029, China; zhaojx@mail.buct.edu.cn; 2 College of Computer Science and Engineering, Beihang University, Beijing 100191, China; liujingchao@buaa.edu.cn

**Keywords:** metal organic frameworks, covalent organic frameworks, photocatalyst, MOF@COF hybrids, Fenton-photocatalytic TCH degradation

## Abstract

In recent years, the response of new porous materials to visible light and their potential applications in wastewater treatment has received extensive attention from the scientific community. Metal Organic Frameworks (MOFs) and Covalent Organic Frameworks (COFs) have been the focus of attention due to their strong visible light absorption, high specific surface area, well-regulated pore structures, and diverse topologies. In this study, a novel MOF@COF composite with a high surface area, high crystallinity, and structural stability was obtained using the covalent bond formation strategy from COF-JLU19 and NH2-MIL-88B(Fe). Under visible light irradiation, the degradation of tetracycline hydrochloride by this material reached more than 90% within 10 min and was completely degraded within 30 min, which exceeded the degradation rate of individual materials. Remarkably, the catalytic activity decreased by less than 5% even after five degradation cycles, indicating good structural stability. The excellent photocatalytic performance of the NM88(DB)@COF-JLU19 hybrids was attributed to the formation of covalent bonds, which formed a non-homogeneous interface that facilitated effective charge separation and promoted the generation of hydroxyl radicals.

## 1. Introduction

In recent years, the widespread misuse of tetracycline hydrochloride (TCH) has led to severe water pollution, posing a threat to humans and the environment over time. As a result, the removal of TCH from contaminated water has received extensive attention [1,2,3,4,5,6,7]. Researchers have developed numerous photocatalysts over the past few decades, but their performance has been less than satisfactory. Over the past few years, efforts have been put into the utilization of cutting-edge two-dimensional crystalline porous materials as photocatalysts [8,9,10]. COFs have garnered increasing attention because of their porous nature, outstanding visible light utilization, and tunable energy band positions in various applications [11,12,13,14,15,16]. However, the quick complexation of photogenerated holes and electrons still limits its photocatalytic activity [17,18,19], a challenge that can be addressed by introducing heterojunctions with other materials [19,20]. MOFs are also popular photocatalysts, with comparable advantages to COFs, as well as various types of metal nodes that can be used as response sites in catalytic reactions [21,22,23].

The combination of both materials maximizes the inheritance of the porous characteristics of both photocatalysts, facilitating mass transfer and significantly promoting photocatalytic reactions [24,25,26]. In 2018, Peng [27] prepared NH_2_-MIL-68@TPA-COF, a nuclear crust structure material, which was the first reported. The composite degraded RhB at a rate 40% higher than NH_2_-MIL-68. Sun [28] introduced an intriguing layered Ti MOFs@Pt@DM-LZU1 hydrophobic photocatalyst, exhibiting excellent photocatalytic performance in the photoreaction of olefins and H_2_. Zou [29] and colleagues used a post-covalent modification strategy to synthesize COF@MOF composites, which demonstrated excellent capabilities in visible-light-catalyzed hydrogen production reactions. The covalent bonding of MOFs and COFs is more favorable for the transport of photoinduced charge carriers [30,31]. However, there is currently limited research on MOF@COF materials for the degradation of tetracycline hydrochloride (TCH).

In this study, we successfully prepared NM88(DB)@COF-JLU19 composite material through covalent bonding, demonstrating the efficient degradation of TCH as a photocatalyst in visible light. Both monomeric materials have been confirmed to hold significant potential in photocatalysis [32,33]. The synthesis involves the aldehyde functionalization of MOFs followed by a reaction between COFs’-NH_2_ groups and MOFs’ aldehyde groups, called a Schiff base reaction, forming covalent bonds. Further exploration of the mass ratio and reaction conditions of the two materials in the experiment revealed that the obtained material exhibited a high degradation efficiency. Specifically, with the addition of a minimal amount of a catalyst, 90% degradation of TCH was achieved within 10 min, reaching nearly complete degradation in 30 min. The speculated mechanism involves a heterojunction formation, with Fe^3+^ in MOFs photoinduced and reduced to Fe^2+^, participating in Fenton-like reactions for TCH degradation. This method has the advantages of fast degradation, non-toxicity, and environmental protection for the friendly and efficient treatment of TCH.

## 2. Materials and Methods

### 2.1. Materials

3,5-Diaminobenzoic acid (DB, 98.0%), 1,3,5-tri-(4-aminophenyl)triazine, 1,3,5-tri-(4-aminophenyl)benzene, and 2-methyl-2-butene were procured from Adamas (Shanghai, China). 1,4-Dioxane (99.0%), hexahydrated iron chloride (FeCl_3_·6H_2_O, 98.0%), and glacial acetic acid were sourced from Damao (Tianjin, China). 2-Aminoterephthalic acid (H_2_ATA, 99.0%), sodium sulfate, and vitamin E were acquired from Macklin (Shanghai, China). Concentrated hydrochloric acid and acetone (99.0%) were bought from Beijing Yili (Beijing, China). Trimethylbenzene was obtained from Guangfu (Tianjin, China). Sodium hypochlorite was purchased from Aladdin (Tianjin, China). *N*,*N*-Dimethylformamide (DMF, 99.5%) was procured from Beijing Chemistry Factory (Beijing, China). Tetrahydrofuran (THF, 99.5%) was sourced from TCI (Tokyo Japan). Sodium hydroxide and sodium thiosulfate were procured from Fuchen Chemical (Tianjin, China). Tetracycline hydrochloride (TCH) was purchased from Mayer (Shanghai, China), and disodium EDTA was sourced from Maya Reagents (Zhejiang, China). The aforementioned reagents were used directly after purchase.

### 2.2. Sample Preparation

#### 2.2.1. Synthesis of NH_2_-MIL-88B(Fe)

In this work, we used the solvothermal method to synthesize NH_2_-MIL-88B(Fe) via a high-pressure hydrothermal synthesis reactor. The detailed procedure was as follows: FeCl_3_·6H_2_O (270 mg), H_2_ATA (180 mg), and DMF (5 mL) were placed in a beaker and then stirred for 10 min. After that, we added 0.4 mL of 2 M NaOH solution and transferred the mixture to a polytetrafluoroethylene inner vessel for 12 h at 100 °C. After the reaction was completed, the product was cooled to room temperature, centrifuged, and washed alternately with deionized water and acetone 5 times. Subsequently, the product was dried at 60 °C to obtain a brown powder product of NH_2_-MIL-88B(Fe) [34], hereinafter referred to as NM88.

#### 2.2.2. Synthesis of DB Anchored NH_2_-MIL-88B(Fe)

To mitigate the impact of steric hindrance during the binding process of MOFs and COFs materials, and to increase the Schiff base reaction sites, thereby enhancing the likelihood of MOFs and COFs binding, before the combination of NM88, an amine-functionalized DB compound was introduced into the reaction mixture of the MOFs to obtain aldehyde-functionalized MOFs. DB was fixed in NM88 via a carboxyl coordination with the metal, and the specific preparation method was the same as NM88. The specific synthesis method was the same as that of NM88. Multiple experimental comparisons showed that the amount of DB added was 10 mg, and the resulting aldehyde-functionalized MOFs were named NM88(DB).

#### 2.2.3. Synthesis of COF-JLU19

The synthesis of COF-JLU19 involves two main steps [32]. The first step is the preparation of imine-linked COF-JLU17, and the specific synthesis steps are as described below: 1,3,5-tris-(4-aminophenyl) triazine (10.6 mg) and 1,3,5-tris-(4-formylphenyl) benzene (11.7 mg) were added to a high-temperature and high-pressure glass flask with a side arm. Then, 0.7 mL of trimethylbenzene, 0.3 mL of 1,4-dioxane, and 0.2 mL of 6 M AcOH were added sequentially. The obtained suspension was ultrasonically mixed for 2 min and then degassed three times through a freeze–pump–thaw process using liquid nitrogen. The container was sealed and reacted at 120 °C for 12 h. After the reaction, the product was cooled to room temperature, filtered, and washed alternately with anhydrous tetrahydrofuran and dichloromethane for 5 cycles. Following that, it was dehydrated in a vacuum at 80 °C for 24 h to obtain a pale-yellow powder product, COF-JLU17.

The second step involves the preparation of amide-linked COF-JLU19, and the detailed synthesis procedures were as follows: 20 mg of COF-JLU17 was put into a container, and 3 mL of 1,4-dioxane was added to obtain a suspension. Then, 0.96 mL of 2-methyl-2-butene, 150 μL of 3.3 M sodium hypochlorite solution, and 51.6 μL of glacial acetic acid were appended sequentially. The above reaction system was kept at room temperature for 12 h away from light, and another 150 μL of 3.3 M sodium hypochlorite solution was added, followed by further incubation for 12 h. The product underwent filtration and was sequentially rinsed with deionized water (10 mL), followed by a 10% sodium thiosulfate solution (10 mL), another round of deionized water (10 mL), and, finally, acetone (10 mL). Subsequently, the resulting material was subjected to vacuum drying at 80 °C for a period of 24 h, culminating in the formation of a yellowish powder, designated as COF-JLU19.

#### 2.2.4. Synthesis of NM88(DB)@COF-JLU19

The preparation method of NM88(DB)@COF-JLU19 is similar to COF-JLU19, but it requires the addition of a specific quantity of NH_2_-MIL-88B(DB) into the precursor material of COF-JLU17. Through multiple experimental comparisons of the influence of NM88(DB) addition on the crystallinity, stability, and catalytic ability of the composite material, the ideal quantity of NM88(DB) was determined to be 30 mg. The resulting NM88(DB)@COF-JLU19(6:4) is a brown powder solid.

### 2.3. Characterization

The structural form and elemental distribution of the samples were analyzed through scanning electron microscopy (SEM; GeminiSEM 300, ZEISS, Shanghai, China) and transmission electron microscopy (TEM; FEI Talos F200x, Thermo Fisher Scientific, Beijing, China) complemented by energy-dispersive X-ray spectroscopy (EDS) for detailed insights. X-ray powder diffraction (PXRD) analyses were conducted using a Rigaku Ultima IV diffractometer (Rigaku, Beijing, China). Additionally, the samples’ chemical bonds were investigated through Fourier transform–infrared spectroscopy (FT-IR) utilizing a Nicolet 6700 spectrometer (FIS, Shanghai, China). Elemental surface distribution of the samples was ascertained using X-ray photoelectron spectroscopy (XPS; Thermo Scientific K-Alpha, Thermo Fisher Scientific, Beijing, China). Thermal gravimetric analysis (TGA/DTG) was performed on the samples using a HITACHI STA200 thermal analyzer (DAZHAN, Shanxi, China), operating under nitrogen gas (50 mL/min) at a heating rate of 10 °C/min across a temperature span of 35 °C to 600 °C. UV-visible diffuse reflectance spectroscopy (UV-vis DRS, Shimadzu, Shanghai, China) was performed using a UV-3600 UV-Vis spectrophotometer. Photoluminescence spectra were collected using an F-700 fluorescence spectrophotometer (Hitachi, Shanghai, China). Electrochemical impedance spectroscopy (EIS), Mott–Schottky analysis (M-S), and photocurrent measurements (I-t) were carried out using an electrochemical workstation (Shanghai Chenhua, Shanghai, China). Copper electrodes and Ag/AgCl electrodes were used as working and reference electrodes, respectively, with a 0.1 M Na_2_SO_4_ electrolyte. The preparation of the working electrode involved the deposition of 200 mL of a suspension (1 mg of the catalyst dispersed in 100 mL water and 10 mL Nafion solution) onto a 1 × 1 cm^2^ ITO glass substrate, followed by drying at 100 °C. Electron Spin Resonance (ESR) spectra were acquired using a paramagnetic resonance spectrometer (Bruker EMXplus, Bruker, Nanjing, China) with DMPO as a spin trap at a concentration of 0.2 M.

### 2.4. Evaluation of Photocatalytic Activity

The photocatalytic efficiency of the composite material was assessed through these steps: 100 mL of a 10 ppm TCH (tetracycline hydrochloride) solution was poured into a beaker. Then, 10 mg of the catalyst was added, followed by stirring in the dark for 30 min to establish adsorption–desorption equilibrium. Subsequently, 20 μL of 30% H_2_O_2_ was incorporated into the mixture. The reaction system was then subjected to illumination under a 300 W xenon lamp (λ > 420 nm) for 30 min. Under the same conditions of guarantee a portion of the solution was taken. The obtained solution was then passed through a 0.22 μm syringe filter, effectively removing any residual catalyst powder.

The concentration of TCH was determined using a UV-Vis spectrophotometer. Specifically, 1 mL of the solution was placed in a quartz cuvette for analysis at a wavelength of 356 nm. These experiments were conducted in triplicate, and the obtained data were averaged to ensure accuracy.

## 3. Results and Discussion

### 3.1. Morphological and Structural Characterization

**Scheme 1:**
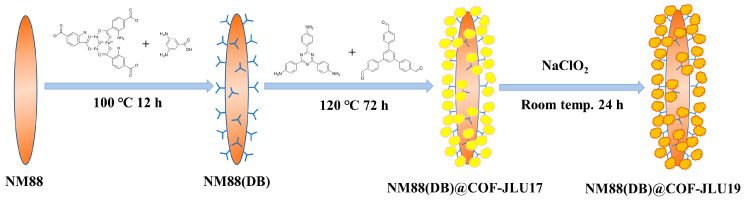
Schematic of the synthesis process for NM88@COF-JLU19 hybrids.

Scheme 1 outlines the preparation procedure of NM88(DB)@COF-JLU19, and all framework materials were prepared following this scheme. NM88 exhibits a brown spindle-shaped morphology. The aldehyde functionalization of NM88 was achieved by introducing DB, connecting aldehyde groups to the NM88 surface. This method strengthens the linkage with the amino groups on the surface of the yellow COF-JLU17. Finally, through oxidation, the brown NM88@COF-JLU19 composite material was obtained.

**Figure 1:**
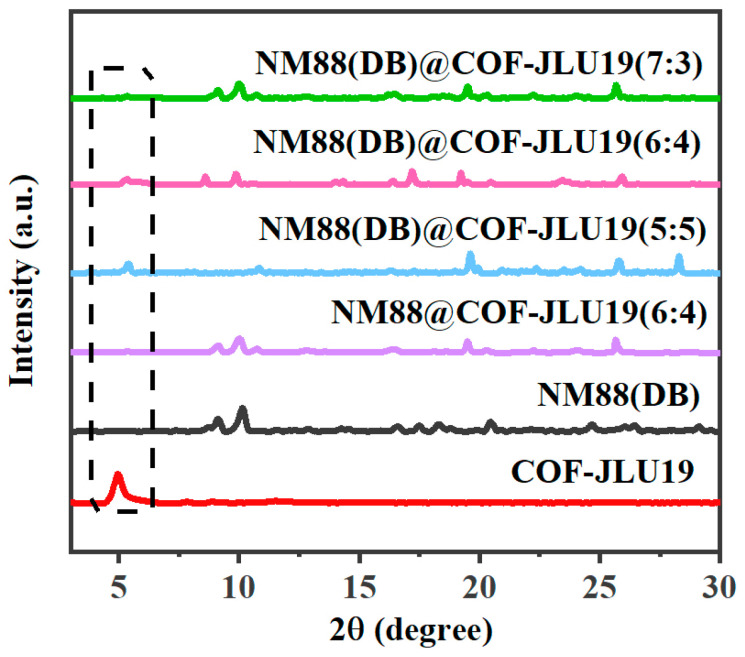
XRD pattern of COF-JLU19, NM88(DB), NM88@COF-JLU19(6:4), and NM88(DB)@COF-JLU19 hybrids.

To validate the presence of crystals, XRD testing was initially conducted. As shown in Figure 1, COF-JLU19 displays a prominent peak at 2θ = 4.96°, along with several less conspicuous broad peaks at 6.96° and 8.36°, aligning with the (100), (110), and (200) planes of a hexagonal lattice, consistent with the literature reports by Masi [32].NM88(DB) displays characteristic NM88 diffraction peaks at 9.24°, 10.33°, 18.52°, and 20.72°, matching the (002), (101), (200), and (202) crystallographic planes [30], indicating that the inclusion of DB has no significant influence on the crystallography of NM88. Due to the relatively small proportion of COF-JLU19 within the composite, the intensity of its diffraction peaks in the material is correspondingly subdued. The diffraction peak positions of the composite material are more similar to those of NM88(DB), which also confirms that NM88(DB) maintained good crystallography and framework stability during the formation of COF-JLU19 [35]. By comparison, it is observable that the material with a mass ratio of NM88(DB):COF-JLU19 = 6:4 has the characteristic peaks of both monomer materials, indicating that a more complete crystalline composite can be obtained at this ratio. When NM88 was doped, COFs peaks did not appear in the obtained composites, and COF-JLU19 can not grow well on MOFs, so the aldehyde formation of MOFs is a crucial step in the formation of composite materials.

**Figure 2:**
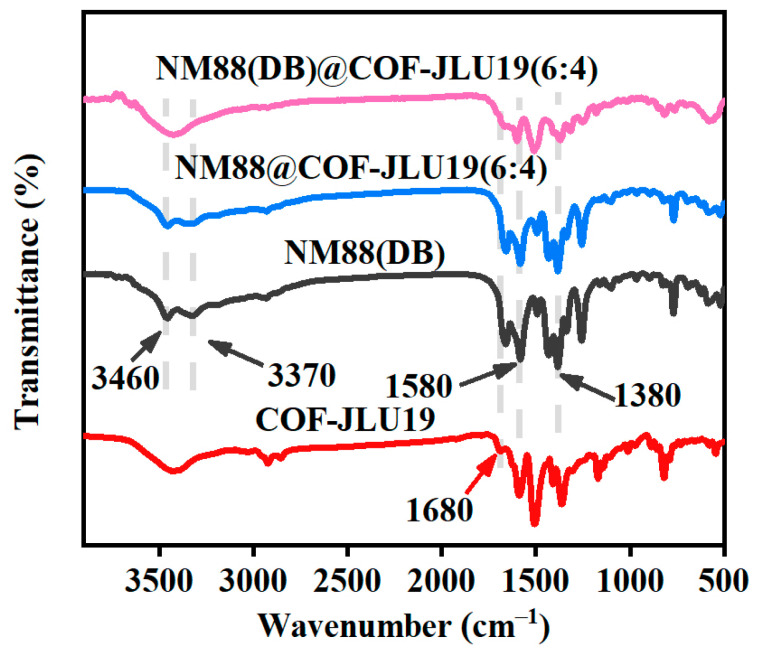
FT-IR image of COF-JLU19, NM88(DB), NM88@COF-JLU19(6:4), and NM88(DB)@COF-JLU19(6:4).

Based on the crystal information obtained through XRD, two composites doped with NM88/NM88(DB) at a ratio of MOF:COF = 6:4, and two monomer materials, were used for infrared testing, and the results are shown in Figure 2. In the aldehyde-functionalized composite material, the peak observed at 1680 cm^−1^ is indicative of the characteristic stretching vibration associated with the amide C=O bond in COF-JLU19 [32]. In comparison to the spectrum of COF-JLU19, this peak exhibits a reduction, suggesting a reaction with the -NH_2_ group of NM88(DB). The peaks at 1380 cm^−1^ and 1580 cm^−1^ are representative of the two C=O bonds in NM88(DB) [36,37]. In the NM88(DB) spectrum, the two peaks at 3370 cm^−1^ and 3460 cm^−1^ are representative of the symmetric and asymmetric characteristic peaks associated with the -NH_2_ [38,39]. Their absence in the composite material’s spectrum further confirms the occurrence of the Schiff base reaction [40], leading to the conclusion that the NM88(DB)@COF-JLU19 (yield: 86%) composite material was successfully synthesized. In the spectra of NM88@COF-JLU19 (yield: 67%), only the characteristic peaks of MOFs materials are observed, signifying that there is no obvious growth of COFs.

**Figure 3:**
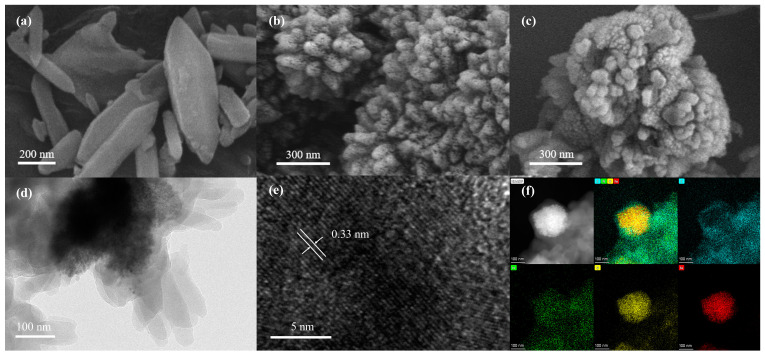
SEM images of NM88(DB) (**a**), COF-JLU19 (**b**), and NM88(DB)@COF-JLU19(6:4) (**c**); TEM images (**d**,**e**) of NM88(DB)@COF-JLU19(6:4) and EDS mapping images (**f**) of NM88(DB)@COF-JLU19(6:4).

The observed morphology under electron microscopy is presented in Figure 3a. NM88(DB) exhibits a spindle-like structure with lengths varying from 300 to 600 nm, with diameters ranging between 70 to 100 nm. The morphology of COF-JLU19 presents a densely aggregated flower-like crystalline structure (Figure 3b). In the morphology of the NM88(DB)@COF-JLU19 (6:4) composite material, it is evident that the flower-like crystalline structure of COF-JLU19 tightly surrounds the spindle-like crystals of NM88(DB), each with an approximate thickness of 100 nm (Figure 3c). As for TEM imaging, it can be seen that the lattice surface spacing of NM88(DB)@COF-JLU19 (6:4) is 0.33 nm, which further explains the high crystallinity of the material. Elemental mapping demonstrates the uniform distribution elements C, N, O, and Fe within the composite material (Figure 3d–f).

**Figure 4:**
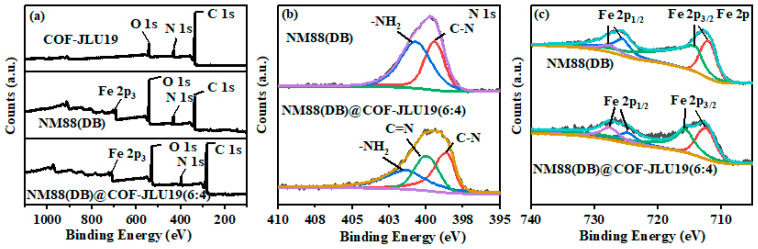
XPS pattern measurements of NM88(DB)@COF-JLU19(6:4): a survey (**a**); N 1s (**b**) and Fe 2p (**c**) binding energy spectra.

The chemical composition and states of the obtained samples were examined using XPS spectroscopy. This spectral analysis confirmed the presence of C, N, O, and Fe elements in both NM88(DB) and the composite material, aligning with findings from the EDS elemental analysis. A further analysis of the chemical states of the elements was performed. As shown in Figure 4b, in the N1s spectrum of NM88(DB), the peaks at 399.5 eV and 401.2 eV correspond to the N atoms in C-N and -NH_2_, respectively [41]. In the N1s spectrum of the composite material, the binding energy of these two peaks is enhanced. This is ascribed to the Schiff base reaction between amino groups and aldehyde groups, following a nucleophilic addition mechanism. In this process, the amine acts as a nucleophilic reagent attacking the aldehyde, resulting in the transfer of electrons from the nitrogen atom of the amine to the carbon atom of the aldehyde. Simultaneously, the peak intensity corresponding to the amino group (-NH_2_) significantly decreases, and the appearance of the C=N peak is observed, providing further evidence of their interaction.

In Figure 4c, the Fe 2p spectrum of NM88(DB) is resolved into four peaks, where Fe 2p_1/2_ and Fe 2p_3/2_ peaks are identified at 725.6 eV and 712.0 eV, respectively, with their associated satellite peaks appearing at 728.3 eV and 716.9 eV, indicative of the presence of Fe(III) in NM88(DB) [42]. Compared to NM88(DB), the composite material exhibits a marginally higher binding energy. In fact, in addition to the amino group electrons in NM88(DB), the electrons associated with Fe(III) are also engaged in the Schiff base reaction, thereby reducing the electron density around Fe(III).

### 3.2. Enhanced Photocatalytic Activity Mechanism

**Figure 5:**
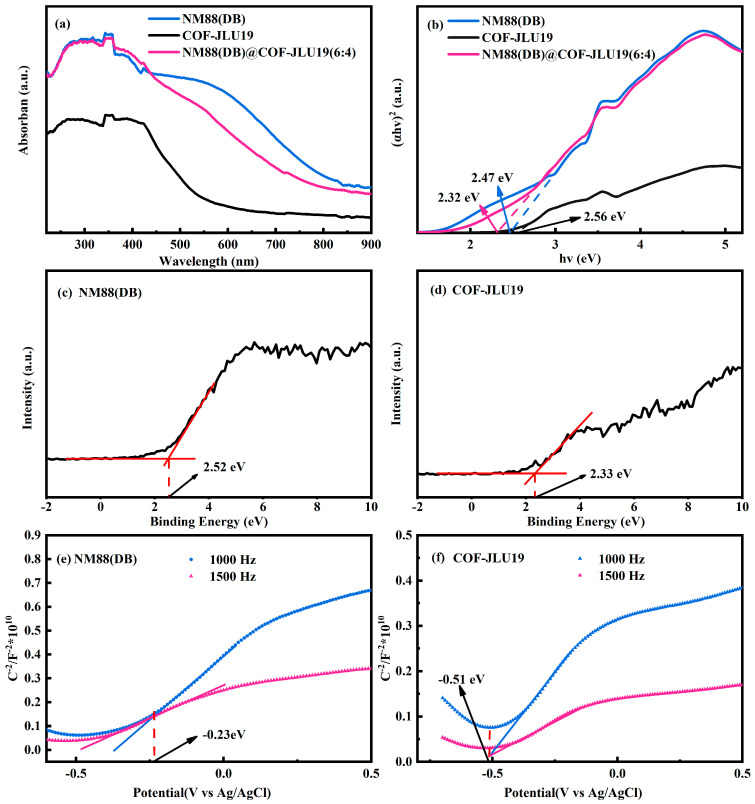
UV-vis DRS spectra (**a**); plots of (Ahv)^2^ as a function of photon energy (**b**) of NM88(DB); COF-JLU19 and NM88(DB)@COF-JLU19(6:4). XPS valence band spectrum (**c**,**d**); typical Mott–Schottky plots (**e**,**f**) of NM88(DB) and COF-JLU19.

To further explore the optical properties of the composite material, optical absorption tests were conducted on several materials using UV-visible spectroscopy. As shown in Figure 5a, the absorption edge of NM88(DB) is situated at 850 nm, while that of COF-JLU19 is at 650 nm. The composite material inherits the favorable absorption characteristics of both individual materials, resulting in a slight extension of the absorption width, which is related to the degree of conjugation in the aromatic system.

Through the calculation of the tangent intercept between (Ahv)^2^ and the photon energy [43], as illustrated in Figure 5b, the estimated bandgap energies (Eg) values for COF-JLU19, NM88(DB), and NM88(DB)@COF-JLU19 were determined as 2.56 eV, 2.47 eV, and 2.32 eV, respectively. Thus, the combination of these two individual materials results in a smaller bandgap width, thereby improving the mobility of photogenerated charge carriers and leading to enhanced optical absorption performance and catalytic capabilities.

The positions of the valence band (VB) and conduction band (CB) for various materials were ascertained using XPS valence band spectra and Mott–Schottky measurements. It is facile to confirm that the VB position of NM88(DB) and COF-JLU19 was approximately 2.52 and 2.33 eV, respectively (Figure 5c,d). According to the Mott–Schottky results (Figure 5e,f), both materials are of the n-type semiconductor nature. In such semiconductors, the flat band potential typically registers as 0.1 eV more positive compared to the conduction band (CB) potential [44]. As shown in Figure 5c,d, the flat band positions of NM88(DB) and COF-JLU19 are −0.23 eV (−0.01 eV vs. NHE) and −0.51 eV (−0.29 eV vs. NHE). As a result, the conduction band positions are 0.09 eV and −0.19 eV, respectively. Integrated with the numerical analysis of the valence and conduction bands, NM88(DB)@COF-JLU19 forms a type II heterojunction, facilitating effective charge separation and excellent conductivity.

By applying Equation (1):

Eg = E_VB_ − E_CB_, (1)

it can be determined that the bandgap widths of NM88(DB) and COF-JLU19 are approximately 2.43 eV and 2.52 eV, respectively. These findings are in significant concordance with the earlier results derived from the UV-visible spectroscopy analysis.

**Figure 6:**
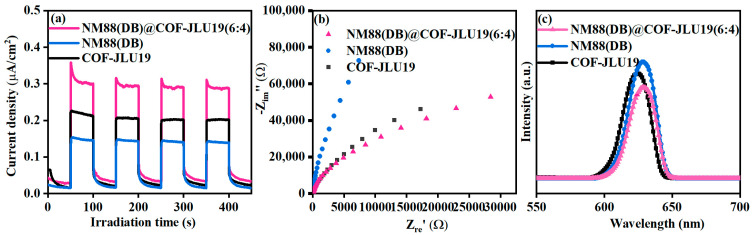
Transient photocurrent response (**a**); EIS Nyquist plots (**b**) and PL spectra (**c**) of NM88(DB), COF-JLU19, and NM88(DB)@COF-JLU19(6:4).

To further analyze the capability of the composite material to generate photogenerated charge carriers, transient photocurrent response tests were conducted. Figure 6a reveals that the photocurrent density of NM88(DB)@COF-JLU19 is twice as high as that of NM88(DB) and 1.5 times that of COF-JLU19, demonstrating the superior photocatalytic ability of the composite material compared to the two individual materials. The electrochemical impedance spectroscopy (EIS) findings presented in Figure 6b demonstrate that the composite material has smaller arc radii compared to the two individual materials, indicating that NM88(DB)@COF-JLU19 has the least interface charge transfer resistance and the most rapid charge transfer rate [45]. The photoluminescence (PL) emission spectra presented in Figure 6c reveal that the composite material exhibits the lowest peak intensity compared to the two individual materials (Solid state, PMT voltage = 300 V, λex = 312 nm, λem = 628.8/629 nm, slits = 5.0/20.0 nm), indicating a lower probability of a photogenerated charge and hole recombination in the NM88(DB)@COF-JLU19 material [46].

### 3.3. Photocatalytic Performance

**Figure 7:**
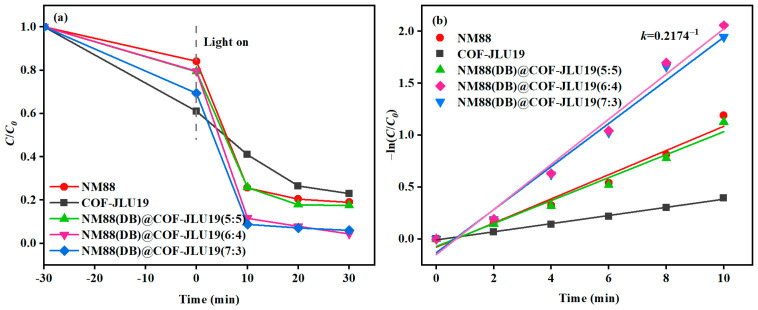
Photo-Fenton degradation of TCH over different materials under visible light irradiation (**a**) and comparison of the degradation kinetics of the degradation of TCH (**b**).

The photocatalytic degradation capabilities of various composite materials and individual materials were compared through the photocatalytic degradation of TCH. As illustrated in Figure 7a, the degradation performance of the composite materials generally surpasses that of the individual materials, as they both achieve more than 95% degradation within 30 min. This confirms that the combination of the two individual materials effectively promotes the progression of the photocatalytic reaction. As the mass ratio of the MOFs material in the composite material increases, the degradation rate changes, with NM88(DB)@COF-JLU19(6:4) exhibiting the fastest degradation rate. This may be related to the thickness of the COFs coating, where overly thin COFs generate fewer photogenerated electrons, and excessively thick COFs obstruct the transfer of these electrons through covalent bonds, as well as interfere with the dispersion and contact of reactants in the catalytic material [47]. Simultaneously, the coating of COFs affects the adsorption capacity of composite materials. The synthesis of COFs monomers and composite materials involves Schiff base reactions. Electron microscopy revealed that MOFs have a larger volume than COFs, with a smaller spatial hindrance between MOFs. Combining the adsorption capacities of various materials in the dark, it is speculated that when a significant amount of MOFs is added, the precursors of COFs disperse directly on the surface of MOFs for reaction. In contrast, with lower MOFs content, COFs precursors, influenced by spatial hindrance, preferentially enter gaps between MOFs and bind with ligands. In such cases, the resulting composite material shows a decreased adsorption capacity due to reduced contact and collision opportunities between highly adsorptive COFs and TCH.

For a more intuitive observation of the rate variation, degradation rate versus reaction time curves and kinetic rate curves were generated (Figure 7b). The order of the rate constants for the two individual materials and composite materials with different ratios is as follows: NM88(DB)@COF-JLU19(6:4) > NM88(DB)@COF-JLU19(7:3) > NM88 > NM88(DB)@COF-JLU19(5:5) > COF-JLU19. At a ratio of 5:5, the rate at which the composite material degrades is lower than that of NM88(DB). This is attributed to the excessively thick COFs. However, as the proportion of NM88(DB) in the composite increases, there is a notable enhancement in its capability to degrade TCH. From the graph, it is evident that the reaction rate of NM88(DB)@COF-JLU19(6:4) is the fastest among the various materials. Table 1 presents the performance data of several newly developed composite materials in the past two years for the degradation of tetracycline hydrochloride. A comparison of catalyst usage and degradation rates indicates that NM88(DB)@COF-JLU19 exhibits excellent performance.

**Figure 8:**
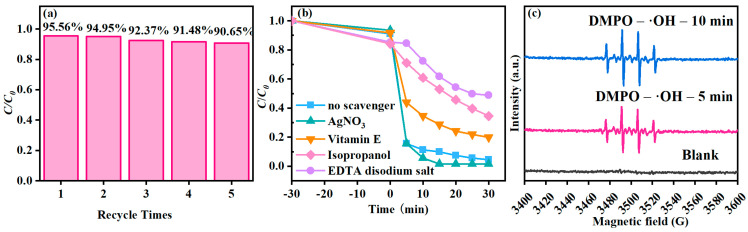
The recyclability and (**a**) the influence of different scavengers (**b**) and ESR spectra of -OH radical species trapped (**c**) by DMPO of NM88(DB)@COF-JLU19(6:4).

**Figure A1:**
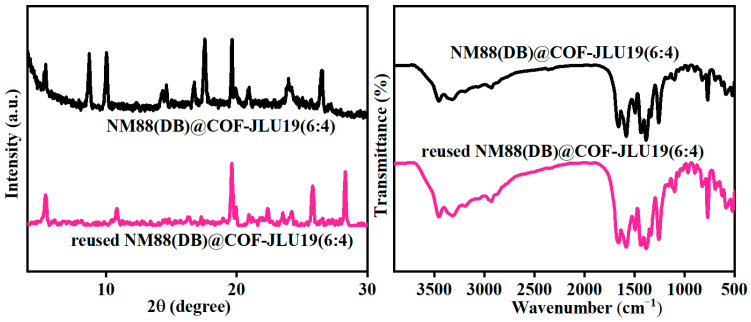
XRD (**a**) and FT-IR (**b**) of NM88(DB)@COF-JLU19(6:4) and reused NM88(DB)@COF-JLU19(6:4).

**Figure A2:**
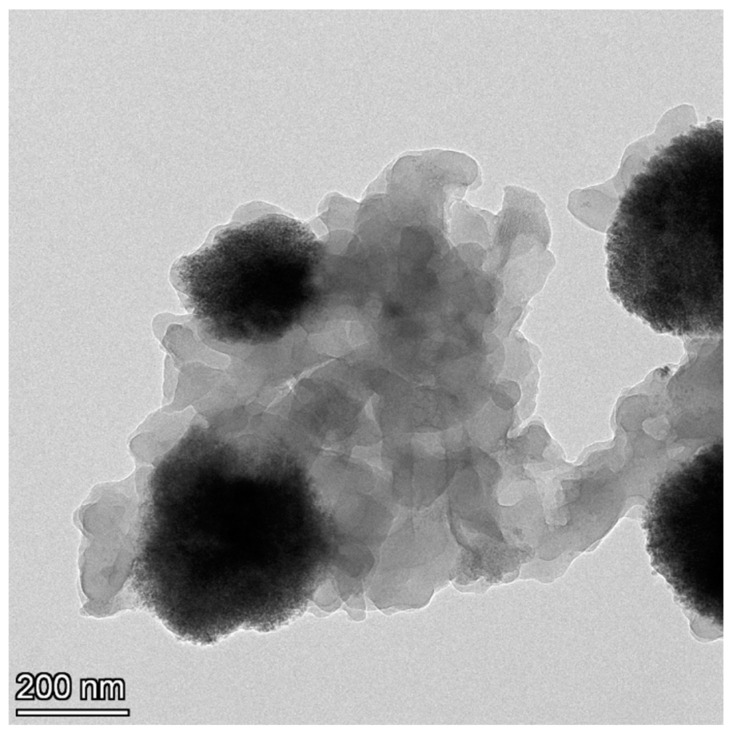
TEM image of reused NM88(DB)@COF-JLU19(6:4).

**Figure A3:**
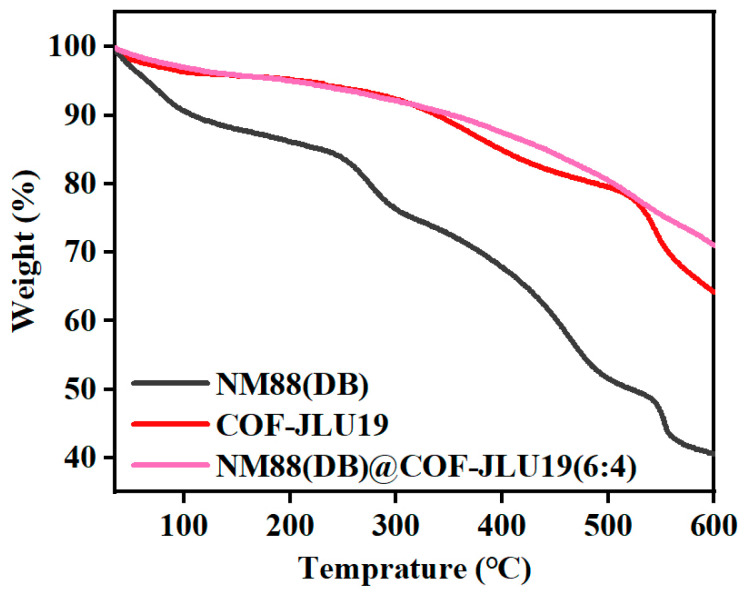
Thermogravimetric analysis of NM88(DB), COF-JLU19, and NM88(DB)@COF-JLU19(6:4).

To further explore the extensive applicability of the synthesized MOF@COF photocatalyst, subsequent photocatalytic degradation experiments were conducted using the optimal photocatalyst, NM88(DB)@COF-JLU19(6:4). To validate the structural integrity and recyclability of the composite material, 5 mg of the composite material was introduced into a 10 ppm TCH solution of 50 mL for photocatalytic degradation experiments. This process was repeated for five cycles, with material collection through centrifugation and subsequent drying in a vacuum oven at 80 °C for 24 h. Remarkably, the NM88(DB)@COF-JLU19 material exhibited a decrease in catalytic activity of less than 5% after five cycles (Figure 8a). Furthermore, the XRD, IR, and TEM test results of the material after cycling were highly similar to those of the freshly prepared catalyst. (Figure A1 and Figure A2) In terms of thermal stability testing, NM88(DB) experienced partial loss of free water and organic solvents before reaching 100 °C due to a lower drying temperature, and the gradual structural collapse was observed to be approaching 250 °C. In contrast, the composite material and COF-JLU19 displayed no noticeable structural degradation before 300 °C (Figure A3), demonstrating its excellent stability.

To investigate the predominant radical groups involved in the process of photocatalytic Fenton-like degradation, radical trapping experiments were undertaken. The concentration of all additives was 10 mM. Figure 8b illustrates that the incorporation of disodium ethylenediaminetetraacetate (EDTA) [53] and isopropanol [54] during the photocatalytic reaction resulted in an obvious decrease in the rate of TCH degradation, which shows that the holes and hydroxyl groups play the main role in the degradation reaction. When AgNO_3_ was introduced into the system, a slight enhancement in the photocatalytic degradation rate of TCH was observed [55]. This suggests that the capture of electrons within the reaction system accelerated the separation rate of photogenerated electrons and holes, with the isolated holes further promoting the progression of the degradation reaction.

Under simulated experimental conditions, electron spin resonance (ESR) was employed to confirm the existence of -OH radicals within the reaction system, as shown Figure 8c. It was noted that a distinct characteristic peak of -OH radicals became evident when deionized water and NM88(DB)@COF-JLU19(6:4) coexisted, and the concentration of free radicals increased with prolonged exposure to light. This indicates that during the degradation reaction, photogenerated holes play a role in directly oxidizing TCH, and they also indirectly contribute to the generation of -OH radicals by oxidizing water.

**Figure 9:**
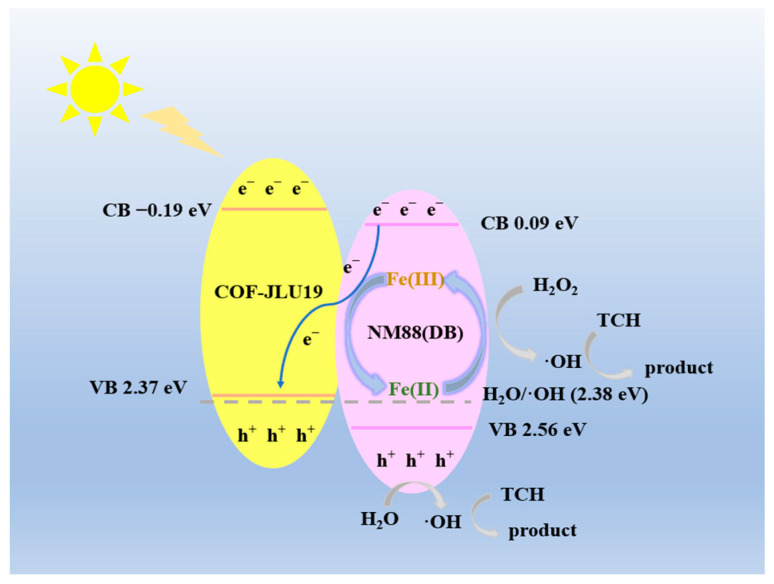
Proposed mechanism of photo-Fenton degradation of TCH in the NM88(DB)@COF-JLU19(6:4)/H_2_O_2_/visible light system.

Combining the previously calculated band structure, Figure 9 illustrates a potential efficient pathway for photocarriers between MOFs and COFs. Generally, the outer COFs exhibit strong visible light absorption capabilities. Photogenerated electrons are conducted through covalent bonds to the enclosed MOFs, which subsequently harness this energy to exhibit strong photocatalytic degradation abilities. In this process, trivalent iron ions in the MOFs obtain electrons and transform H_2_O_2_ in the system into -OH radicals. Simultaneously, the holes generated in the valence band of COFs demonstrate potent oxidation capabilities to convert water into -OH radicals. These various radical species cooperate to collectively oxidize and degrade tetracycline hydrochloride.

## 4. Conclusions

In conclusion, we developed a simple and effective post-covalent modification strategy to create a covalently linked composite material through the aldehyde functionalization of NH_2_-MIL-88B, followed by tightly encapsulating it with a defined thickness of COF-JLU19. A photoelectrochemical analysis demonstrated that the covalent bonds between the two materials act as mediators for charge transfer, markedly enhancing the separation efficiency of electron–hole pairs. As one might expect, the resulting composite material exhibits excellent stability and superior capability for degrading tetracycline hydrochloride under visible light conditions. Under visible light irradiation, NM88(DB)@COF-JLU19 (6:4) at a concentration of 0.1 g/L demonstrates a remarkable degradation efficiency, exceeding 90% within 10 min for 10 ppm TCH hydrochloride. This performance far surpasses that of individual materials. Importantly, even after five degradation cycles, the catalytic activity only decreases by less than 5%. This study establishes NM88(DB)@COF-JLU19 as a stable and efficient photocatalyst, offering promising prospects for the treatment of tetracycline (TCH) pollutants in wastewater via the photocatalytic Fenton-like process.

## Figures and Tables

**Table 1:** Comparison of the degradation rate of TCH by different catalysts.

Catalyst	C (mg/L)	Catalyst Dosage ^1^	k × 10^−3^ (min^−1^)	Ref.
MIL-125 (Ti)-derived TiO_2_	300	15	1.03	[48]
NiAl_2_O_4_/g-C_3_N_4_	300	15	0.50	[49]
TiO_2_/PPy/NiCoP	400	40	0.12	[50]
BOC/NMLT-5	600	24	0.40	[51]
Fe0.25Cu0.75(BDC)@DE	500	25	0.31	[52]
NM88(DB)@COF-JLU19	100	10	3.17	This work

^1^ Catalyst dosage: ratio of the amount of catalyst to the amount of pollutant.

## Data Availability

Data are contained within the article.
